# Clear cell sarcoma of the kidney misdiagnosed as mesoblastic nephroma: a case report and review of the literature

**DOI:** 10.3332/ecancer.2013.311

**Published:** 2013-04-24

**Authors:** Samin Alavi, Maliheh Khoddami, Mohammad Kaji Yazdi, Paria Dehghanian, Sadaf Esteghamati

**Affiliations:** 1 Shahid Beheshti Medical University, Tehran, Iran; 2 Mofid children’s hospital, Hoseinieh Ershad, Tehran, Iran

**Keywords:** clear cell sarcoma of the kidney, sternum, frontal bone, mesoblastic nephroma, bone metastasis

## Abstract

Clear cell sarcoma of the kidney (CCSK) is a rare renal neoplasm of paediatrics, making up about 3% of all renal tumours in paediatrics, with a high tendency for developing bone metastasis. A seven year-old boy was referred to our clinic with two firm, large masses over the manubrium of the sternum and right frontal area, which pathologically were confirmed as a metastatic CCSK. The patient had a history of a renal mass three years earlier, for which radical nephrectomy had been performed, and histopathologic diagnosis was compatible with mesoblastic nephroma. Thus, no further investigation and therapy had been applied for the patient. CCSK is a rare but malignant and aggressive paediatric renal tumour, with a high tendency for developing distant bone metastases, leading to its poor prognosis. CCSK could be misdiagnosed as several other renal tumours such as mesoblastic nephroma, and thus CCSK should be taken carefully into consideration in the diagnosis of renal tumours.

## Introduction

Clear cell sarcoma of the kidney (CCSK) is a rare renal neoplasm of paediatrics, comprising about 3% of all paediatric renal tumours [1, 2]. Formerly, CCSK was classified as Wilms’ tumour until 1970, when it was defined as a separate and independent entity [3]. CCSK was initially recognised because of its tendency to metastasise to the bone, leading to its poor prognosis (survival rate <30%), despite currently available chemotherapy regimens and radical surgery [1, 4]. The clinical presentation bears a resemblance to Wilms’ tumour, including abdominal mass, abdominal pain, and gross haematuria. CCSK is more common in male individuals and usually occurs as solitary renal mass in two- to three-year-old children [1]. CCSK has a high tendency for developing bone metastasis through its course, and thus it was previously called a ’bone-metastasizing renal tumour of childhood’ [5]. The skeleton and skull are the most common sites of bone metastases and are associated with poor outcome and high mortality [6]. As these tumours are radiologically indistinguishable from Wilms’ tumour [7] and the prognosis is very poor [1, 4, 6], the clinical entity should be kept in mind, so that the diagnosis and treatment are not postponed. We report a rare case of CCSK presenting at an older age with two metastatic masses in the sternum and frontal bones; the patient had previously undergone nephrectomy following a diagnosis of mesoblastic nephroma.

## Case presentation

A seven-year-old boy was referred to our clinic because of swelling over his sternum and the right side of the frontal bone. On physical examination, there was a firm, large mass measuring 3 cm × 4 cm lying over the manubrium of the sternum and another bony mass measuring 4 cm × 5 cm over the right frontal area. The patient had a history of right renal mass three years earlier, for which radical nephrectomy had been performed. The histopathologic evaluation of the renal mass had been reported as mesoblastic nephroma. Thus, no further investigation and therapy had been applied for the patient at that time.

**Figure 1:**
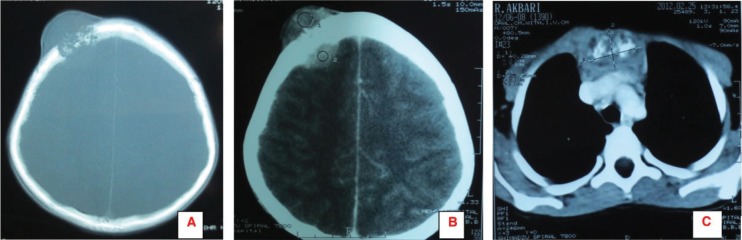
A, B: Brain CT scan shows an expansile bone lesion in the right frontal bone, invading the surrounding soft tissues. C: Chest CT scan shows a heterogeneous mass in the manubrium of the sternum composed of soft tissue and bone components.

Following his referral to our clinic, the patient underwent a thorough examination including brain, chest, and abdominopelvic computed tomography (CT), which were normal except for two bony mass lesions. A brain CT scan showed an expansile bone lesion in the right frontal bone, invading the surrounding soft tissues (Figures 1A, B). Similarly, a chest CT scan showed a heterogeneous mass in the manubrium of the sternum composed of soft tissue and bone components, in favour of metastasis (Figure 1C). Whole-body scintigraphy with 99mTc-MDP showed an increased uptake of radiotracer in the frontal bone and the manubrium of the sternum. The patient was scheduled for excisional biopsy of the sternum mass. Histopathological examination revealed fibromuscular tissue infiltrated with round tumour cells with small to moderate amounts of cytoplasm and high mitotic activity accompanied by vascular invasion, which was consistent with a small round cell tumour with sarcomatous pattern (Figure 2A, B). Re-evaluation of pathological specimens at the time of nephrectomy was carried out which was in favour of CCSK and revealed a misdiagnosis of mesoblastic nephroma in the earlier report. Immunohistochemistry on the specimen of the sternal mass was performed, revealing negative results for WT-1 and positive results for CD-99 in favour of CCSK (Figures 3A, B).

The patient was diagnosed with relapsed, metastatic CCSK accounting for stage IV. Chemotherapy with Ifosfamide, Carboplatin, and Etoposide (ICE protocol) was started, and due to the lack of favourable response after three courses, doxotubicin and cyclophosphamide were added. Six months later, he had additional active bone lesions in the left humerus and ischium. He was scheduled to receive radiotherapy over his involved bones and also considered as a candidate for autologous stem cell transplantation.

## Discussion

In this report, we presented a case of metastatic CCSK that was previously misdiagnosed as mesoblastic nephroma, and thus no further action had been taken. The patient presented with two bone masses in the sternum and frontal bone (three years after the renal mass), which were diagnosed to be metastatic CCSK. 

Wilms’ tumour is considered the most common malignant (renal) tumour of paediatrics, comprising 85% of all malignant renal tumours in this age group. Mesoblastic nephroma is the second most prevalent malignant paediatric renal tumour (5%), followed by clear cell sarcoma (4%), rhabdoid tumours (2%), and other rare tumours (2%) [8]. CCSK, being rare, is the most frequently misdiagnosed paediatric renal tumour. The morphological diversity and scarceness of appropriate diagnostic markers along with a rare presentation are responsible for misdiagnosis of this tumour [9]. About 5% of patients with CCSK present with metastases, and thus the prognosis is very poor. In addition, the metastatic disease might mimic other benign conditions and lead to delays in diagnosis and treatment [10]. The histopathological characteristics of CCSK include cells that are plump and ovoid or spindle-shaped with pale cytoplasm and fairly uniform round to oval, and often vesicular, nuclei with finely dispersed chromatin, inconspicuous nucleoli, and infrequent mitotic figures [11]. Several histological variants have been recognised in addition to the classic type, including myxoid, sclerosing, cellular, epithelioid, palisading, storiform, spindle cell and anaplastic types. Most of the tumours have more than a single histology pattern, and thus the diagnosis could be controversial [1]. The bone masses in our study tested negative for WT-1 and positive for CD-99, which is in favour of CCSK. Immunohistochemistry could be considered a useful tool for distinguishing CCSK from other childhood renal tumours. It has been reported that CCSK is positive for vimentin and CD-99 [1, 12, 13]. Bonadio and Perlman [13] performed an analysis of 61 CCSK tumours, demonstrating nerve growth factor receptor (NGFR) staining in all tested cases. It is also well established that CCSK is uniformly negative for cytokeratin, Mic-2, S100, neural markers, desmin, and WT-1 [1, 14]. 

Mesoblastic nephroma is another rare malignant renal tumour that was formerly considered a benign tumour until metastatic cases were reported [15]. Currently, it is suggested that complete resection of mesoblastic nephroma (nephroureterectomy) is the treatment of choice for this tumour, and it is also an independent predictor of prognosis [16]. Most patients with mesoblastic nephroma are diagnosed within the first three months of life. There is also a male predominance [15]. In our patient, the primary tumour was diagnosed and treated at three years of age, when mesoblastic nephroma was very improbable. In other words, three years of age is the common age for CCSK. The histopathology also differs dramatically, especially the nuclei (round to oval, and often vesicular, nuclei) that are characteristic of CCSK.

## Conclusion

In conclusion, CCSK is a rare but malignant and aggressive paediatric renal tumour, with a high tendency for developing distant bone metastases, leading to poor prognosis. CCSK could be misdiagnosed as several other renal tumours such as mesoblastic nephroma; thus, CCSK should be taken into consideration in the diagnosis of renal tumours.

## Figures and Tables

**Figure 2A:**
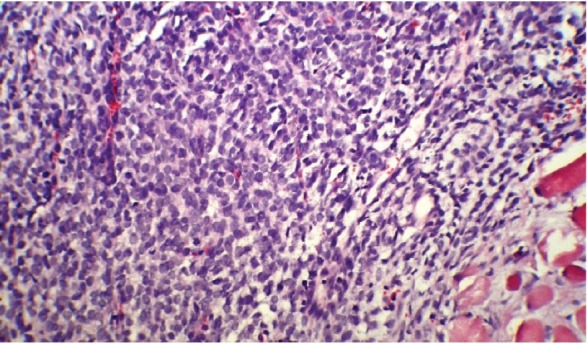
A low-magnification (10x) pathology of the sternal mass showing fibromuscular tissue infiltrated with round tumour cells.

**Figure 2B:**
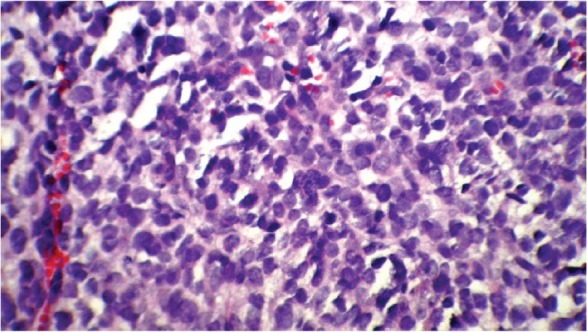
A high-magnification (40x) pathology shows round cells with small to moderate amounts of cytoplasm and high mitotic activity accompanied by vascular invasion.

**Figure 3A:**
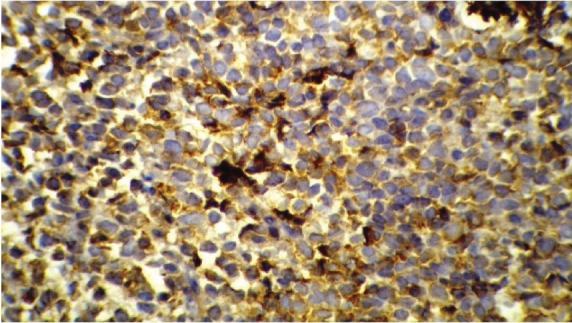
Immunohistochemical staining---positive for CD 99.

**Figure 3B:**
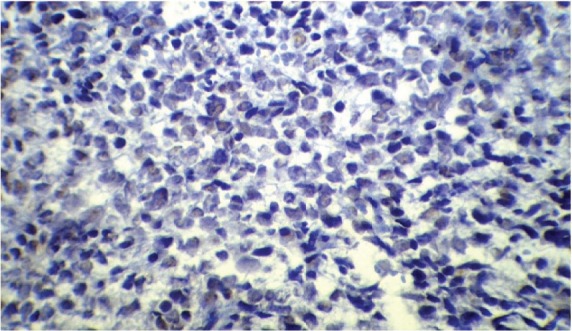
Immunohistochemical staining---negative for WT-1.

## References

[1] Argani P, Perlman EJ, Breslow NE, Browning NG, Green DM, DíAngio GJ (2000). Clear cell sarcoma of the kidney: a review of 351 cases from the National Wilms Tumor Study Group Pathology Center. Am J Surg Pathol.

[2] Radulescu VC, Gerrard M, Moertel C, Grundy PE, Mathias L, Feusner J (2008). Treatment of recurrent clear cell sarcoma of the kidney with brain metastasis. Pediatr Blood Cancer.

[3] Kidd JM (1970). Exclusion of certain renal neoplasms from the category of Wilms tumor. Am J Pathol.

[4] Marsden HB, Lawler W (1980). Bone metastasizing renal tumour of childhood. Histopathological and clinical review of 38 cases. Virchows Arch A Pathol Anat Histol.

[5] Marsden HB, Lawler W (1978). Bone-metastasizing renal tumour of childhood. Br J Cancer.

[6] Sotelo-Avila C, Gonzalez-Crussi F, Sadowinski S, Gooch WM, Pena R (1985). Clear cell sarcoma of the kidney: a clinicopathologic study of 21 patients with long-term follow-up evaluation. Hum Pathol.

[7] Hadley GP, Sheik-Gafoor MH (2010). Clear cell sarcoma of the kidney in children: experience in a developing country. Pediatr Surg Int.

[8] Miniati D, Gay AN, Parks KV, Naik-Mathuria BJ, Hicks J, Nuchtern JG (2008). Imaging accuracy and incidence of Wilmsí and non-Wilmsí renal tumors in children. J Pediatr Surg.

[9] Boo YJ, Fisher JC, Haley MJ, Cowles RA, Kandel JJ, Yamashiro DJ (2009). Vascular characterization of clear cell sarcoma of the kidney in a child: a case report and review. J Pediatr Surg.

[10] Lowe LH, Isuani BH, Heller RM, Stein SM, Johnson JE, Navarro OM (2000). Pediatric renal masses: Wilms tumor and beyond. Radiographics.

[11] Watts KE, Hansel DE, MacLennan GT (2011). Clear cell sarcoma of the kidney. J Urol.

[12] Kural AR, Onal B, Ozkara H, Cakarir C, Ayan I, Agaoglu FY (2006). Adult clear cell sarcoma of the kidney: a case report. BMC Urol.

[13] Bonadio J, Perlman EJ (2008). Immunohistochemical analysis of 61 clear cell sarcomas of the kidney for a panel including NGFR and CD99. Mod Pathol.

[14] Balarezo FS, Joshi VV (2001). Clear cell sarcoma of the pediatric kidney: detailed description and analysis of variant histologic patterns of a tumor with many faces. Adv Anat Pathol.

[15] England RJ, Haider N, Vujanic GM, Kelsey A, Stiller CA, Pritchard-Jones K Mesoblasticnephroma: a report of the United Kingdom Childrenís Cancer and Leukaemia Group (CCLG). Pediatr Blood Cancer.

[16] Furtwaengler R, Reinhard H, Leuschner I, Schenk JP, Goebel U, Claviez A (2006). Mesoblasticnephromaóa report from the Gesellschaft fur PädiatrischeOnkologie und Hämatologie (GPOH). Cancer.

